# Novel research on nanocellulose production by a marine *Bacillus velezensis* strain SMR: a comparative study

**DOI:** 10.1038/s41598-020-70857-7

**Published:** 2020-08-26

**Authors:** Samia S. Abouelkheir, Marwa S. Kamara, Salma M. Atia, Sara A. Amer, Marina I. Youssef, Rana S. Abdelkawy, Sherine N. Khattab, Soraya A. Sabry

**Affiliations:** 1 Marine Microbiology Laboratory, Marine Environment Division, National Institute of Oceanography and Fisheries (NIOF), Kayet Bay, El-Anfushy, Alexandria, Egypt; 2 Industrial Microbiology and Applied Chemistry (IMAC) Program, Faculty of Science, Alexandria University, Alexandria, Egypt; 3 Department of Chemistry, Faculty of Science, Alexandria University, Alexandria, 21321 Egypt; 4 Botany and Microbiology Department, Faculty of Science, Alexandria University, Alexandria, 21321 Egypt

**Keywords:** Biotechnology, Microbiology

## Abstract

Bacterial nanocellulose (BNC) is a nanofibrillar polymer that possesses unique characteristics such as high chemical purity, mechanical strength, flexibility, and absorbency. In addition, different bacterial strains can form nanocellulose (NC) in multiple shapes and sizes. This study describes the first report of a marine *Bacillus* strain that is able to synthesize NC. The strain identified as *B. velezensis* SMR based on 16S rDNA sequencing, produced highly structured NC, as confirmed by X-ray diffraction (XRD) and Scanning Electron Microscopic Analysis (SEM). In Hestrin-Schramm (HS) medium, *B. velezensis* SMR produced twice the quantity of BNC in comparison to the reference strain, *G. xylinus* ATCC 10245. The ability of *B. velezensis* SMR to produce NC using different industrial waste materials as growth media was tested. Growth in *Ulva* seaweed extract supported a 2.5-fold increase of NC production by *B. velezensis* SMR and a threefold increase in NC production by *G. xylinus* ATCC 10245. As proof of principle for the usability of NC from *B. velezensis* SMR, we successfully fabricated a BNC-based polyvinyl alcohol hydrogel (BNC-PVA) system, a promising material used in different fields of application such as medicine, food, and agriculture.

## Introduction

Bacterial nanocellulose (BNC), an extracellular produced structure, is considered a highly desirable biomaterial due to its superior qualities in comparison to other cellulose-containing structures. Compared to plant cellulose, nanofibril network of biocellulose possesses high water retaining capacity, degree of polymerization, chemical purity, high crystallinity, in vivo biocompatibility to be used as a scaffold in tissue engineering, and excellent mechanical properties^1,2^.

BNC production has been reported in both Gram-negative bacteria such as *G. xylinus*, *Agrobacterium, Achromobacter, Aerobacter, Azotobacter, Pseudomonas*, and *Rhizobium*, and Gram-positive bacteria such as *Sarcina*^3^. Among the different BNC-producing bacteria, *G. xylinus* is the most commonly studied species^4^. Synthesis of BNC is a complex process involving polymerization of glucose monomers and secretion of the complex cellulose structures to the external environment to create a three-dimensional microfibril and nanofibril network. During the fermentation process, bacteria either move freely in the media or attach to cellulose fibers, producing a highly swollen gel structure^5^. Purification of NC from the culture medium involves the removal of bacterial cells and collection of the cellulose matrix from the cultural medium. This is a crucial step to ensure the quality of BNC and can be performed either by repeated washing using a hot sodium hydroxide solution, followed by water until reaching a neutral pH or by other methods, such as gamma radiation^6^.

While BNC-production by several bacterial species have been reported, it has never been shown that members of the genus *Bacillus* were able to produce NC. *B. velezensis* is a Gram-positive bacterium that has been extensively studied for its ability to induce plant growth-promotion and its use in biocontrol^7^. *B. velezensis* type strain was isolated from the river bank of Vélez in Málaga in Southern Spain and was reported to grow at a pH range between 5.0 and 10.0 and at temperature between 15 and 45°C^8^. *B. velezensis* strain 11‑5 was isolated previously from marine environment^9^. Recent research suggests that marine microorganisms have superior characteristics compared to their terrestrial counterparts. They produce multiple secondary metabolites with the potential for using seawater in place of freshwater for fermentation. This is very important especially in arid zones like the Middle East. Therefore, the combination of marine biomass, marine microorganism and seawater has a potential for a greener biomaterials production^10^.

*B. velezensis* produces diverse metabolic intermediates, which include antibiotics, enzymes, phytohormones, iron chelators, antioxidants, growth-promoters, and antitumor agents^11–13^. Although *B. velezensis* is classified as a heterotypic synonym of *B. amyloliquefaciens* subsp. *plantarum* FZB42T based on DNA-hybridization values greater than 84%^14,15^, strains within the species still show distinct genomic characteristics that may warrant different genomospecies^16^.

BNC is a notably versatile biomaterial and has been used in a broad range of industrial, technology, and biomedical applications. Moreover, BNC is biodegradable and nontoxic with mechanical and structural properties that can be exploited in numerous applications such as paper products, electronics, acoustics, and biomedical devices. BNC is a type of dietary fiber Generally Recognized As Safe (GRAS) food, approved for marketing by the US Food and Drug Administration (FDA) in 1992^17^. Hence, it can be used in food packaging and fabrication of paper and textiles^18^. In the field of biomedicine, it can be used in delivery of dermal drugs^19^, recuperation of burned skin, wound dressing, and artificial blood vessels. Moreover, due to its high water-retention capacity and its similarity to auricular cartilage in terms of mechanical strength and host tissue response, it is a promising biomaterial for auricular cartilage tissue engineering^3^.

The conventional method for producing BNC using glucose as carbon source is a high cost process and cannot be produced on large industrial scale. Therefore, production of BNC using cheap industrial waste as carbon, nitrogen and energy sources is required and has been extensively studied^20–23^. Coffee cherry husk extract (a byproduct from the coffee processing)^21^ was used as less expensive source of carbon and a lot of other wastes like waste from fruit processing^22^. The use of such materials could enhance the sustainability of BNC yielding as well as diminish environmental pollution linked with the disposal of industrial waste.

This work describes for the first time the production of nanocellulose from a marine *B. velezensis* SMR strain using biomass of the alga *Ulva* sp as sole carbon source in seawater based medium. In addition, the physical and structural properties of the BNC were investigated. The BNC produced was applied in BNC-based polyvinyl alcohol hydrogel (BNC-PVA) system.

## Results and discussion

**Figure 1:**
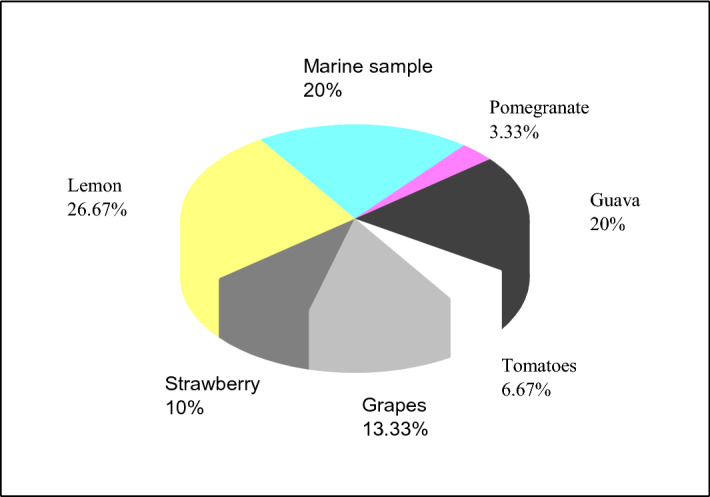
Percentage of bacterial isolates forming thin mesh layer on HS broth after static growth for 7 days at 30 °C and showing positive results with Fehling test.

Nanocellulose production by isolated bacteria was assessed by the formation of complex mesh structure on the surface of the cultural media during static growth^24^, followed by Fehling test to confirm the carbohydrate nature of the membranes. Positive results were found among 33 isolates out of 44 recovered from different sources (Fig. 1).

**Figure 2:**
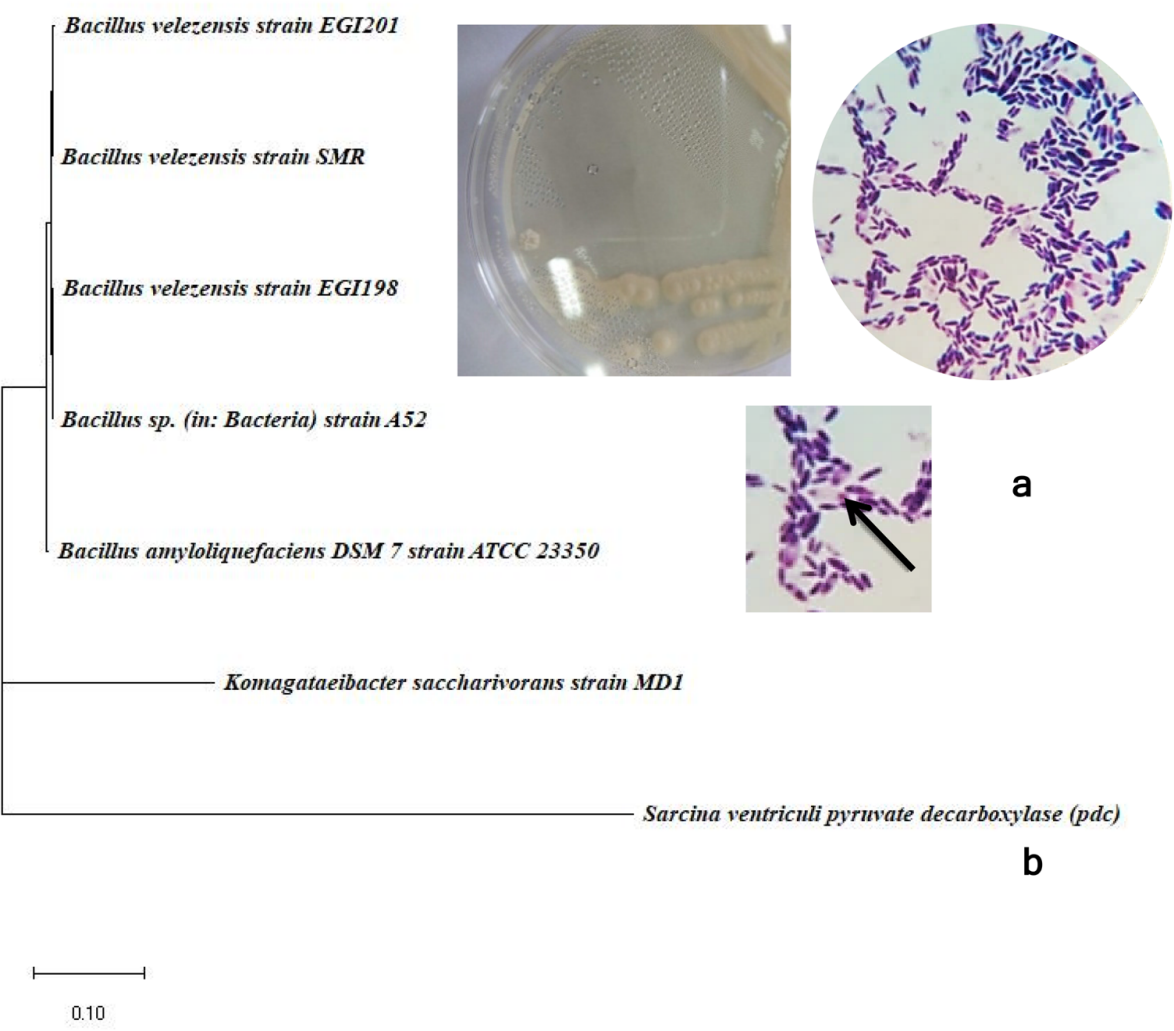
Colony and cell morphology of *B. velezensis* SMR, endospore is shown by an arrow (**a**), phylogenetic tree based on the 16SrDNA sequence of *B. velezensis* SMR and closely related bacterial species (**b**).

An isolate that produced notably a thicker membrane was characterized as endospore Gram + ve rod shaped forming mucoid creamy colonies on agar plates (Fig. 2a). All other BNC- producing isolates were Gram-negative. The Gram-positive isolate was identified as *Bacillus velezensis* based on 16S rDNA sequence that showed 100% similarity to *Bacillus velezensis* strains EGI198 (accession number MN704466.1), and EGI201 (MN704467.1). Accordingly, this isolate was identified as *Bacillus velezensis* SMR and the sequence was deposited in the National Center for Biotechnology Information (NCBI) GeneBank under the accession number MT232963 (https://submit.ncbi.nlm.nih.gov/subs/?search=SUB7182479). The phylogenetic tree of the 16S rDNA of *Bacillus velezensis* SMR and its relation to the available sequences on the NCBI GenBank database is illustrated in Fig. 2b.

While *B. velezensis* SMR produced NC, interestingly, different *B. velezensis* isolates were shown to possess the genes encoding putative lignocellulolytic enzymes and were able to efficiently degrade lignocellulosic, cellulosic and hemicellulosic materials^25,26^. Moreover, it has been shown that *B. velezensis* strain 157 is able to degrade various agro-industrial byproducts including soybean meal, wheat bran, sugarcane bagasse, wheat straw, rice husk, maize flour and maize straw utilized in biofuel production. *B. velezensis* strains were also investigated for their ability to depolymerize various types of lignocelluloses into fermentable sugars^27^. Nair et al*.*^27^ isolated *B. velezensis* ASN1 that could synthesize cellulase used in food, textile, animal feed, petroleum, waste management, biosurfactant, and pulp/paper industries.

**Figure 3:**
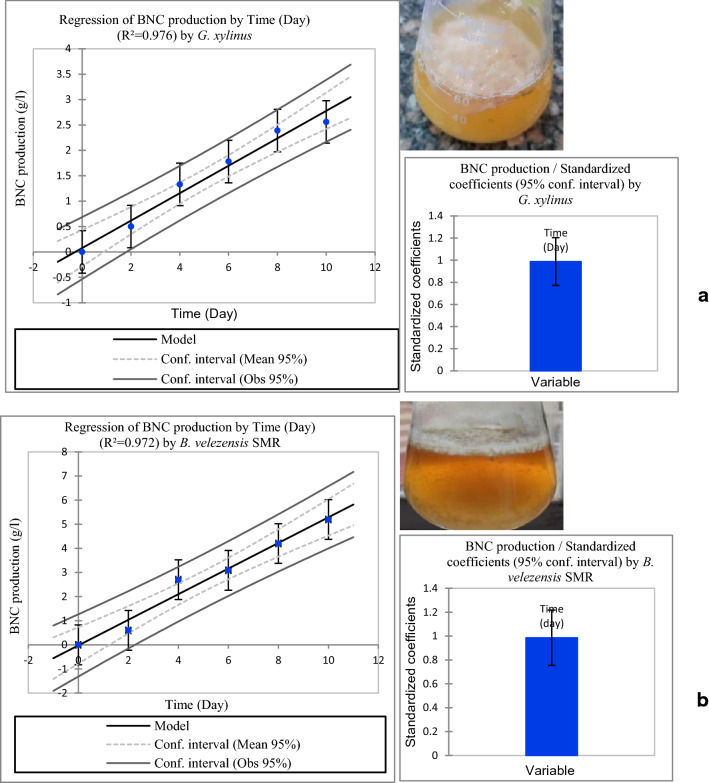
Graphs showing thin layer of BNC formed at the air liquid interface in liquid cultures and the linear regression of the effect of incubation time on BNC production by *G. xylinus* (**a**) and *B. velezensis* SMR (**b**). Data are presented as means ± SE. Values were significant, when P < 0.05 and non-significant, when P > 0.05. There is a significant positive relationship between BNC production and time for *G. xylinus* (r(4) = 0.970, P < 0.001with SE = 0.078) and *B. velezensis* SMR (r(4) = 0.965, P < 0.001 with SE = 0.084) when n = 4.

With respect to BNC production, *B. velezensis* SMR was compared to the reference strain *G. xylinus*. Both strains were cultured in HS broth at pH 5 and 30 °C under static growth conditions for ten days. The produced BNC appeared as a white highly flexible membrane with artificial-looking leather (Fig. 3a,b). The nanocellulose product was purified and estimated as dry weight. It was noticed that after 10 days of incubation, *B. velezensis* SMR produced an average of 5.2 g/L NC compared to 2.6 g/L biosynthesized by *G. xylinus*. The residual glucose concentration in g/L was measured every two days for ten days to monitor the glucose consumption (see Supplementary Fig. S1). In the case of *G. xylinus*, a major problem in using glucose as a carbon source is the formation of by-products including gluconic acid that decreases medium pH, which inhibits BC production^28^. Several options to tackle this problem would be to optimize glucose feeding strategy and/or to use alternative carbon sources that do not trigger the production of by-products^28^.

### Fourier-transform infrared spectroscopy (FTIR)

**Figure 4:**
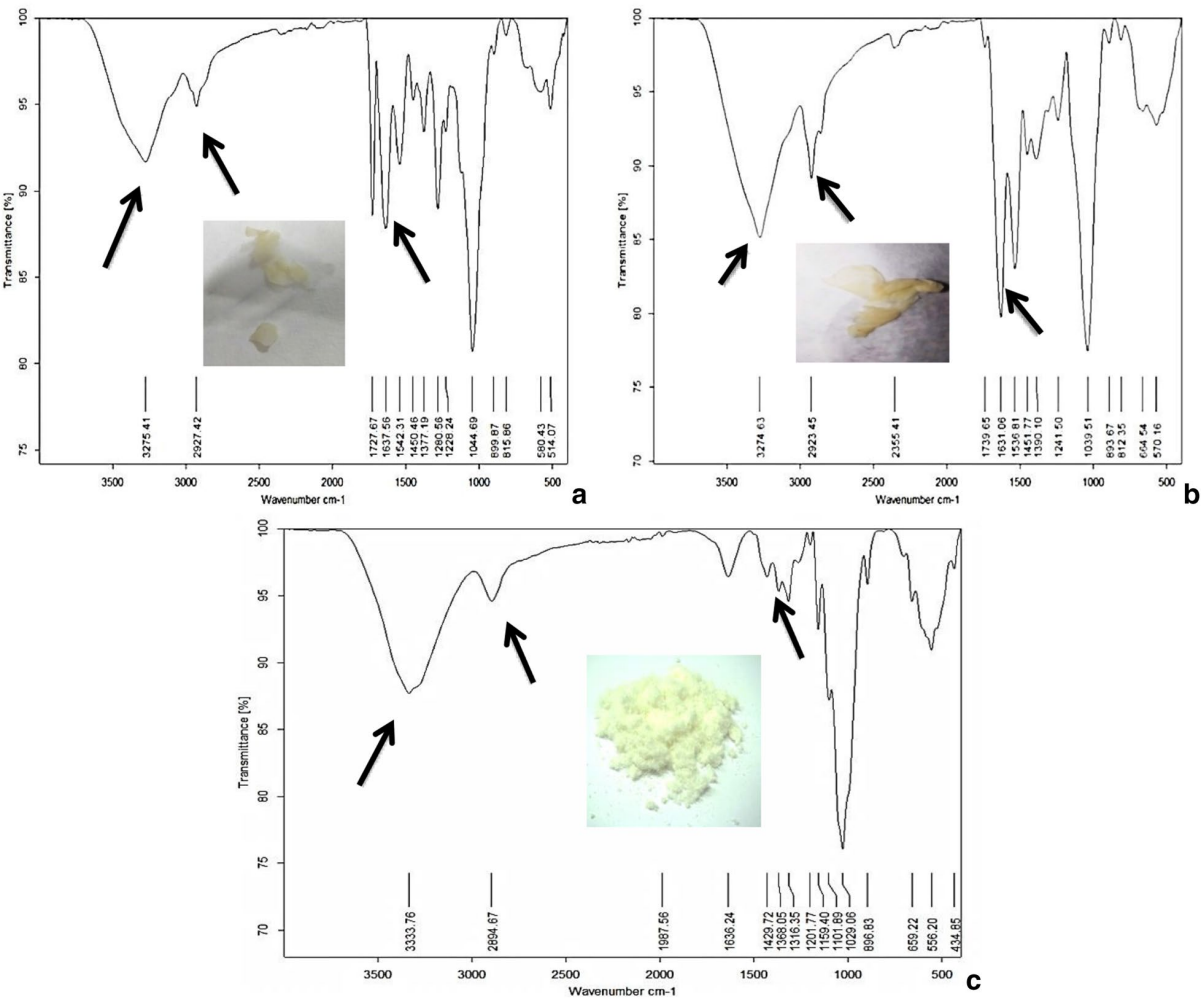
FTIR spectra of NC from *G. xylinus* (**a**), *B. velezensis* SMR (**b**), and rice husk (**c**), arrows refer to some characteristic peaks, absence of one peak in c is due to the induced chemical changes as a result of plant cellulose chemical treatments.

BNC samples of *G. xylinus* and *B. velezensis* SMR were analyzed by FTIR in comparison to plant cellulose (derived from dried rice husk) (Fig. 4). The observed hydroxyl groups (OH) in C1, C3 and C6 mainly contribute to the formation of various kinds of inter- and intra-molecular hydrogen bonds and reflect the tendency of all NC samples to be hydrophilic. It has been shown that hydrogen bonds formation between cellulosic fibers and other materials gives rise to great benefits for the research on all other aspects of natural fibers and related materials as stated by Fan et al*.*^29^. The FTIR spectra and the peak positions of the major IR bands were compared to data in the literature^30–34^ (Table 1).

**Table 1:** FTIR analysis of NC: wavenumber related regions in comparison between *G. xylinus* (a), *B. velezensis* SMR (b), and rice husk (c).

Peak wavenumber of *G. xylinus*-NC ${\mathrm{cm}}^{-1}$ (a)	Peak wavenumber of *B. velezensis* SMR-NC ${\mathrm{cm}}^{-1}$ (b)	Peak wavenumber of rice husk-NC ${\mathrm{cm}}^{-1}$ (c)	Bonds
3275	3275	3334	OH stretching^30,31^
2927	2923	2835	C–H symmetric stretching^31,32^
1638	1631	1636	OH bending of absorbed water^30,31^
1450	1452	–	HCH and OCH in plane bending vibration^31,33^
1377	1390	1368	CH bending in-the-plane^31,34^
1314	1317	1316	CH_2_ rocking, OH plane deformation^31,34^
1045	1040	1029	C–O stretching^31,34^

### Scanning electron microscopy (SEM)

**Figure 5:**
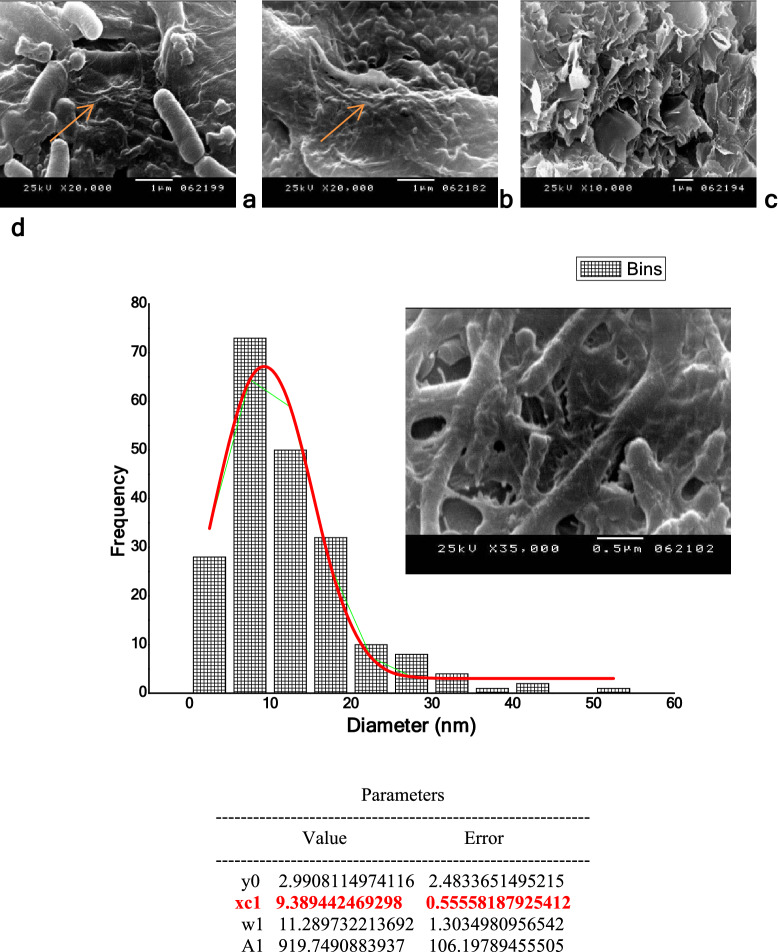
SEM images of *G. xylinus* (**a**) and *B. velezensis* SMR (**b**) showing cells embedded in BNC fibers. Nanocellulose sheet formed after 10 days of incubation (**c**), and size distribution curve of *B. velezensis* SMR-BNC fibers with average size 9.389 nm (**d**).

SEM micrographs of BNC (Fig. 5a,b) show the compact nanocellulose network structure consisting of a random assembly of fibrils (see Supplementary Fig. S2). Both purified BNC pellicles exhibited a reticulated structure consisting of ultrafine nanofibrils (Fig. 5c) (see Supplementary Fig. S3). Nanocellulose fibers created by *B. velezensis* SMR had a diameter range from 1 to 60 nm with mean particle size xc1 = 9.389 nm as obviously observed from the nanoparticle size distribution curve (Fig. 5d). Previous study concerning the production of BNC excreted by *G. xylinus* in HS medium declared that the obtained fibers diameter was in the range of 1 nm to 120 nm with an average of 60 nm^35^.

### X-ray diffraction

**Figure 6:**
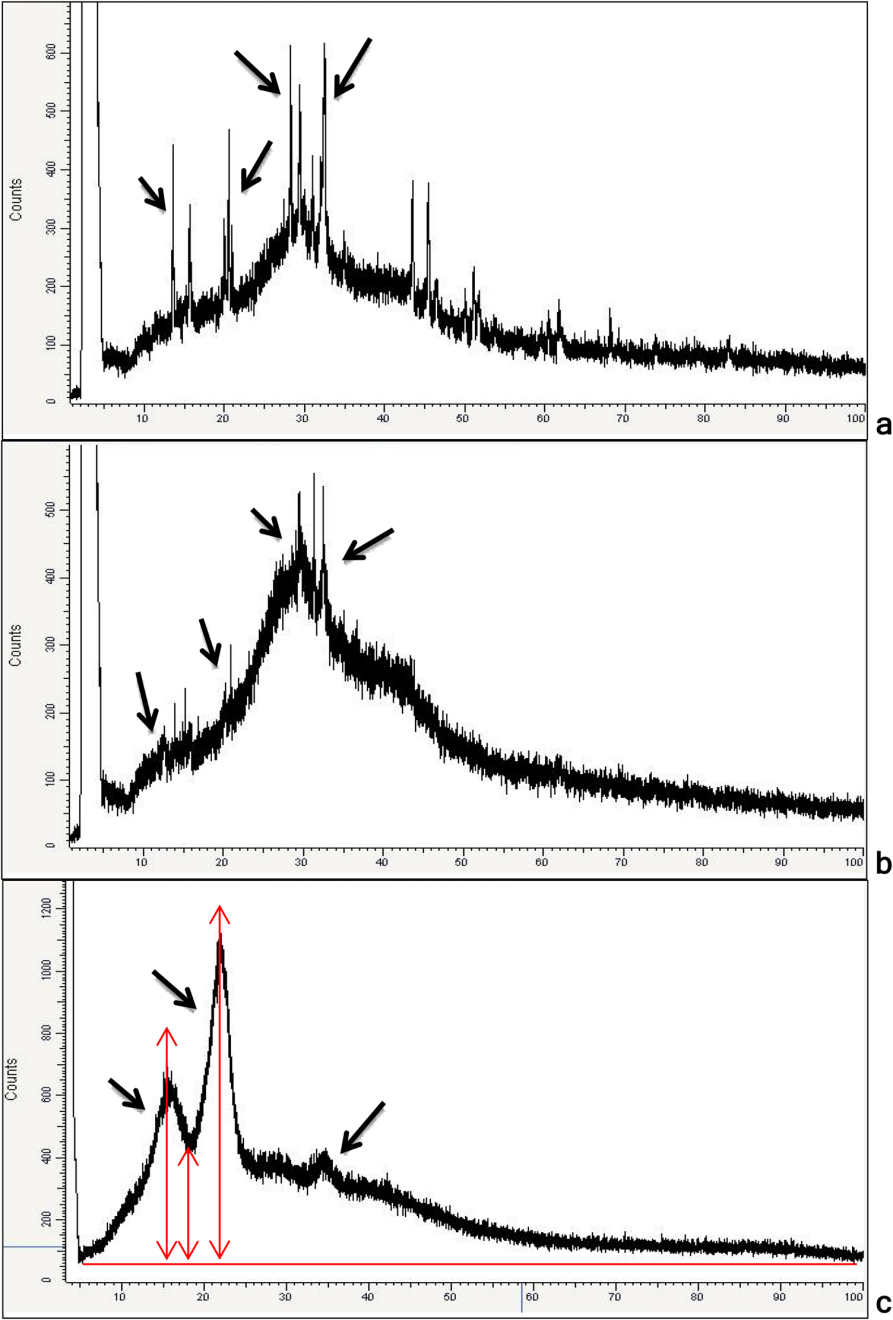
X-Ray Diffractogram of NC of *G. xylinus* (**a**), *B. velezensis* SMR (**b**), and rice husk (c), arrows refer to the CrI (%) calculation from the ratio of the height of the maximum (I_max_) and the height of the minimum (I_am_) (**c**).

X-ray diffractometer (XRD) was applied to determine the grain size and crystallinity of the produced NC. XRD analysis of *G. xylinus*-BNC (Fig. 6a) showed four characteristic peaks at Bragg’s angle 2θ (13.006°–15.026°) with peak position at 14.016°, (19.005°–22.015°) 20.025°, (27.034°–29.003°) 28.014°, and (31.013°–33.023°) 32.508°. The crystallite sizes calculated from Scherrer equation Eq. (1) were 42, 42, 85, and 43 nm, respectively.

The X-ray diffractogram of *B. velezensis* SMR-BNC (Fig. 6b) showed four signature peaks at Bragg’s angle 2θ (11.006°–13.046°) with peak position at 12.006°, (13.046°–16.803°) 15.036°, (21.005°–32.003°) 30.003°, and (31.003°–33.003°) 32.578° in accordance with Fan et al*.* (2016)^35^. These observed peaks correspond to the crystal planes <110> , <110> , <200> and <004> , respectively. The grain sizes calculated from Scherrer equation Eq. (1) were 42, 42, 17, and 43 nm, respectively with a major peak observed at 30.003° with 17 nm.

The crystallinity of rice husk fibers was typical to cellulose with three well-defined crystalline peaks around 2θ (11.996°–18.995°) with peak position at 16.006°, (18.995°–24.994°) 21.994°, and (32.003°–36.002°) 34.406° (Fig. 6c). These characteristic peaks for cellulose are corresponding to the lattice planes <110> , <200> and <004> , respectively. The grain sizes calculated from Eq. (1) were 21, 28, and 22 nm, respectively^30^. According to Eq. (2), the Crystallinity Index (CrI) of BNC produced by *G. xylinus*, *B. velezensis* SMR, and rice husk-NC was about 80, 85, and 67.44%, respectively (Table 2). In a previous study, the CrI of BNC produced by *G. xylinus* CICC No. 10529 was approximately 65%^35^, which was lower than BNC produced by *G. xylinus* 80%, and *B. Velezensis* SMR 85%.

**Table 2:** XRD analysis of NC of *G. xylinus* (a), *B. velezensis* SMR (b), and rice husk (c).

XRD analysis	*G. xylinus*-NC	*B. velezensis* SMR-NC	Rice husk-NC
Characteristic peaks at Bragg’s angle 2θ	14.016°, 20.025°, 28.014°, 32.508°	12.006°, 15.036°, 30.003°, 32.578°^35^	16.006°, 21.994°, 34.406°
Crystallite sizes (CS)	42, 42, 85, and 43 nm, respectively	42, 42, **17**, and 43 nm, respectively	21, 28, and 22 nm, respectively^30^
Crystallinity Index (CI %)	80%	85%	67.44%

### Thermogravimetric analysis (TGA)

**Figure 7:**
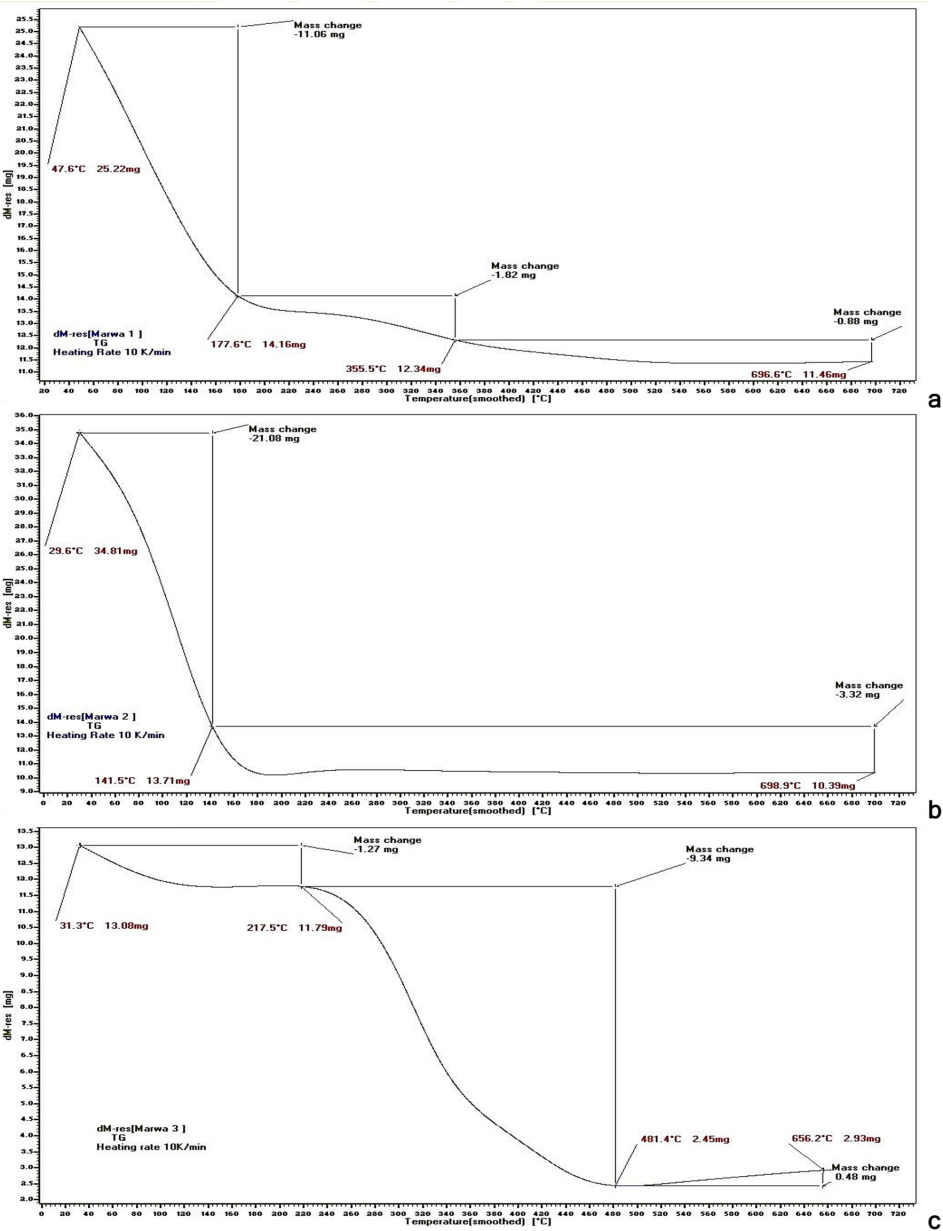
TG curves show the thermal degradation behavior of NC from *G. xylinus* (**a**), *B. velezensis* SMR (**b**), and rice husk (**c**).

Thermogravimetric analysis was performed to study the thermal degradation behavior of the three NC samples (Fig. 7). The primary alteration was attributed to the vaporization of water content due to the hydrophilic feature of the cellulosic fibers, which appeared for BNC samples from *G. xylinus* (a), *B. velezensis* SMR (b), and rice husk-NC (c) at 177.6 °C (43.85% wt loss), 141.5 °C (60.61%) and 217.5 °C (9.86%), respectively. The weight loss was dependent on the primary moisture content of the examined materials. The NC samples a, b, and c exhibited a major degradation process at 540 °C (54.56% wt loss), 170 °C (70.15%) and 481.4 °C (81.30%) leaving 45.44%, 29.85% and 18.7%, respectively, as remaining mass residues (Table 3) (see Supplementary Fig. S4a–c).

**Table 3:** Water content calculated according to Eq. (3) and total loss in NC weight produced by *G. xylinus* (a), *B. velezensis* SMR (b), and rice husk (c).

Sample	Water content (%)	Total loss of weight (%)
*G. xylinus*-NC	43.85	54.56
*B. velezensis* SMR-NC	60.6	70.15
Rice husk-NC	9.86	81.3

BNC of *G. xylinus* was estimated to be stable at temperature 540 °C before complete degradation, while the stability of *B. velezensis* SMR**-**BNC and rice husk-NC was until 170 °C and 481.4 °C, respectively. This shows that marine *B. velezensis* SMR-BNC had less stability but higher water content which makes it a good biomaterial for various biomedical applications (e.g. wound dressing). In comparison to plant cellulose, BNC showed excellent physical properties with respect to mechanical stability, tensile strength, and crystallinity^36,37^.

### Formation of bacterial nanocellulose /polyvinyl alcohol (BNC/PVA) hydrogel

**Figure 8:**
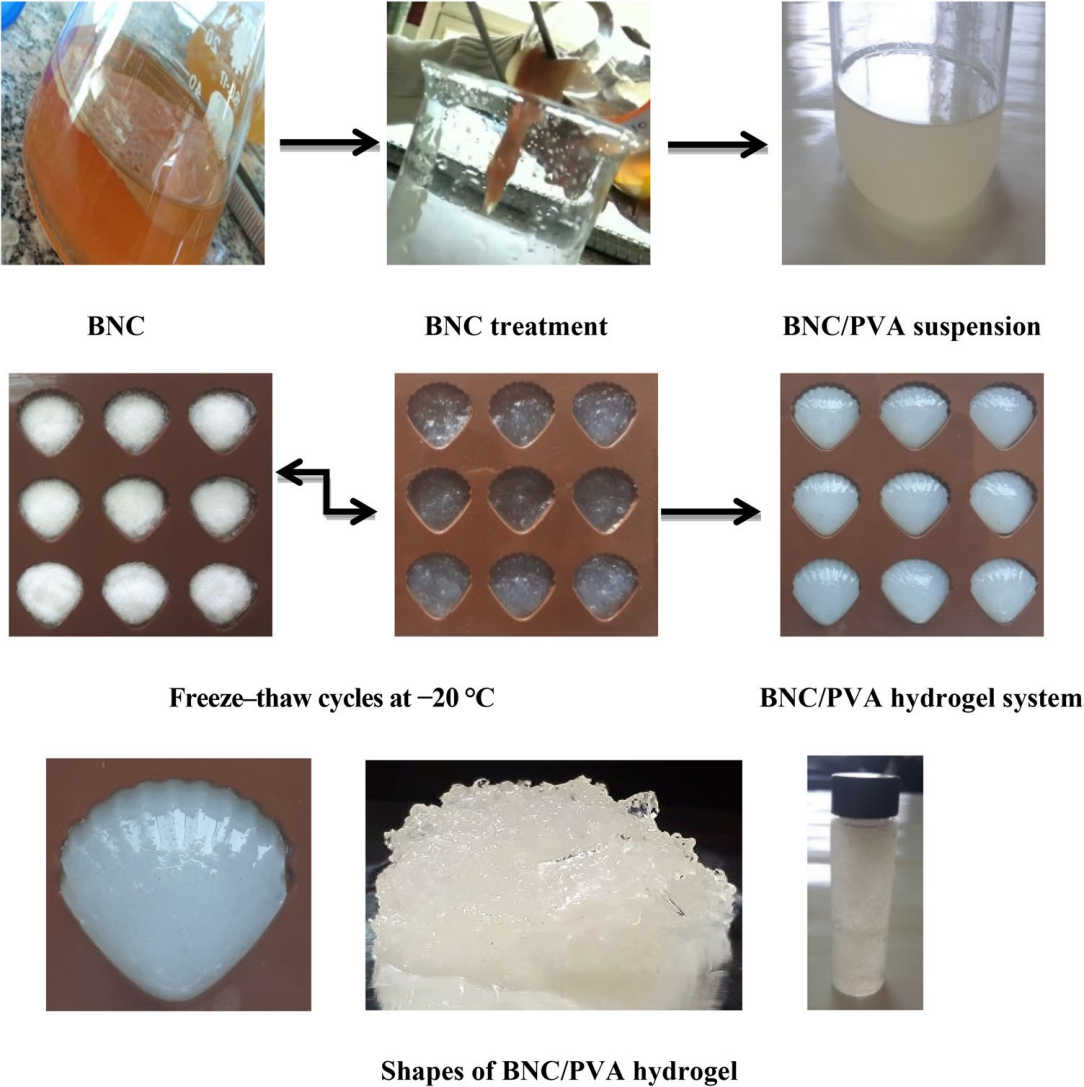
A schematic laboratory diagram demonstrating series of steps for the production of *B. velezensis* SMR-BNC, treatment and combination with PVA, formation of BNC/PVA hydrogel system after freeze–thaw cycles at − 20 °C, and finally controlling the hydrogel system shape.

Polyvinyl alcohol (PVA) hydrogel containing BNC was prepared by direct dispersion of the nanofibers in an aqueous PVA solution (20%) as illustrated in the schematic diagram (Fig. 8). Polyvinyl alcohol (PVA) is a water-soluble polymer that has been extensively investigated because of its good biocompatibility and mechanical properties. PVA solution can form rigid hydrogel through freeze–thaw cycles. During freezing, the PVA chains interact to form a matrix which acts as physical cross-links, maintaining the insolubility of the material in water.

Similar to the BNC-PVA hydrogel system fabricated in this work, Millon et al*.*^38^ produced a PVA hydrogel reinforced with BNC with mechanical properties analogous to cardiovascular tissues. Castro et al*.*^39^ percolated BNC with PVA; the BNC-PVA systems were subjected to the freeze–thaw technique to promote the physical cross-linking of PVA. Li et al*.*^40^ investigated the effect of the amount of freezable bound water on the BNC hydrogels and physically cross-linked PVA. There was a significant increase in the amount of freezable bound water with more than 20% PVA. In this state, the water molecules were more strongly attached to the hydrogel, which hindered the loss of water and crack formation under compression.

### Production of BNC using industrial and agricultural wastes

In this study, we used several industrial and agricultural wastes, which included sugarcane bagasse, molasses, rice bark, rice husk, palm fronds, paper waste, and dried algal biomass. The dry weight of BNC produced by the two examined strains using various wastes in g/L was assessed (Table 4). The use of the green alga *Ulva* sp. in seawater based medium was the most promising substrate yielding 13 and 9.6 g/L with *B. velezensis* SMR and *G. xylinus*, respectively. Previous studies successfully used various wastes for BNC production^28,35,41^. Sugarcane molasses was used in seawater-based media for production of bioethanol using a marine yeast strain^42,43^. Using molasses, BNC levels were estimated as 5.6 and 5.1 g/L using *G. xylinus*, and *B. velezensis* SMR, respectively. These amounts are much higher than that obtained by *G. saccharivorans* MD1 3.9 g/L after incubation for 168 h^31^. Moosavi-Nasab and Yousefi^20^ studied the feasibility of using low quality date syrup a fruit largely produced in the hot arid regions of Southwest Asia and North Africa, for the production of BNC using *G. xylinus*.

**Table 4:** Mean values of BNC produced (g/L) of *B. velezensis* SMR and *G. xylinus*.

Substrate (g/L)		*B. velezensis* SMR			*G. xylinus*		
		BNC (g/L)	Yield (%)	Productivity (g/L/time)	BNC (g/L)	Yield (%)	Productivity (g/L/time)
Glucose (control)	20	5.2	100	0.52	2.6	100	0.26
Rice husk	20	3.8	73.07	0.38	2.9	111.5	0.29
Sugarcane	20	2.3	44.23	0.23	2.1	80.76	0.21
Palm fronds	20	3.9	75	0.39	4.0	153.8	0.40
Rice bark	20	3.7	71.15	0.37	3.4	130.7	0.34
Dried *Ulva* sp.	20	13.0	250	1.3	9.6	369.2	0.96
Molasses (mL)	20	5.1	98.07	0.51	5.6	215.3	0.56
Paper	20	4.4	84.61	0.44	5.0	192.3	0.50

Yield was calculated as % based on the total glucose (20 g).

## Conclusion

Bacterial nanocellulose is a promising material in nearer future for its unique properties and wide range of application in industry, technology, biotechnology and medicine. We herein report an economical approach for the production of nanocellulose by a marine *Bacillus* species using alga biomass as growth medium, which is a cheap substrate widely found in the seashore of the Mediterranean Sea. Moreover and to reduce the environmental footprint of the bio-production of NC, we demonstrate the potential of using seawater instead of distilled water in the growth medium. To the best of our knowledge, this is the first report to show that a bacterial isolate belong to the genus *Bacillus* is able to synthesize NC. In addition, we highlight the ability of *B. velezensis* SMR to produce NC at a comparable or significantly higher level in comparison to *G. xylinus*, which is traditionally used as a reference strain for NC production. Morphological, physical and structural analysis of NC produced by *B. velezensis* SMR show good mechanical stability, tensile strength, and crystallinity. As proof of principle for the usability of NC from *B. velezensis* SMR, we successfully fabricated a BNC-based polyvinyl alcohol hydrogel (BNC-PVA) system, a promising material used in different fields of application such as medicine, food, and agriculture.

## Materials and methods

### Samples

Samples of acidic rotten fruits such as pomegranate, tomatoes, grapes, strawberries, guava, and lemon utilized for the isolation of NC-producers were collected from markets, juice-bars, and used after incubation at room temperature in sealed glass jars for 1 week. Mediterranean seawater was also utilized as a source of isolation.

### Bacteria

The marine bacterium used throughout this study was isolated from seawater and identified using 16SrDNA sequence as *Bacillus velezensis* SMR. The reference strain, *Gluconoacetobacter xylinus* ATCC 10245 (now known as *Komagatabacter xylinus*) was obtained from Microbiological Resources Center (Cairo MIRCEN), Egypt.

### Culture medium and growth conditions

*Hestrin-Schramm (HS) medium* in g/L seawater (20 d-glucose, 5 yeast extract, 5 peptone, 2.7 disodium phosphate, 1.15 citric acid, and 30 agar, pH 5.0)^44^ was used for isolation and BNC production. Medium was autoclaved at 121 °C for 20 min. Glucose was autoclaved separately from the other medium components. pH was adjusted to 5.0 using buffer system (35.0 mL 0.1 M-citric acid and 65.0 mL 0.1 M-trisodium citrate mixed).

### Screening for nanocellulose producers

Isolation was performed using serial dilution technique^45^. One milliliter of each sample was used to inoculate agar plates of HS medium and incubated for 24–48 h at 30 °C. Colonies were picked up and purified. A loopful of each isolate was inoculated into 50 mL of the same medium in100 mL conical flasks and incubated statically at 30 °C for 7 days. Flasks with white pellicle on the surface of the growth medium were selected for further analysis. The pellicles formed at the air–liquid interface were collected by centrifugation at 6,000 rpm for 10 min, treated with 1 N NaOH at 80 °C for 15 min, and washed 3 to 4 times with distilled water^6^. Fehling test was used after acidic hydrolysis to confirm the carbohydrate nature of the products to facilitate selection of exopolysaccharide producers.

### Plant cellulose extraction

Plant cellulose was extracted from rice husk by alkali treatment and purified to remove lignin and hemicellulose according to Johar et al*.*^46^.

### Fourier-transform infrared spectroscopy (FTIR)

FTIR analysis was performed to compare BNC and plant cellulose powder. FTIR analysis was examined using Bruker Tensor 37 FTIR Spectroscopy. Spectral range was from 4,000 to 500 cm^−1^, and the signal obtained at 1 cm^−1^ resolution^47^.

### Bacterial identification

Based on FTIR analysis, bacteria that expressed high BNC level was selected and identified by amplification and sequencing of the 16S rDNA. Bacterial cells collected from overnight HS culture were used for DNA isolation^48^. The PCR reaction was carried out using the universal 16S rRNA primers 27F: 5′-AGA GTT TGA TCM TGG CTC AG-3′ and 1492R: 5′-CGG TTA CCT TGT TAC GAC TT-3′. The amplification was done as follows: initial denaturation at 95 °C for 5 min followed by 35 cycles of 94 °C, 55 °C and 72 °C for one minute each, and a final extension at 72 °C for 10 min. The PCR product was analyzed on 1% agarose gel-electrophoresis, purified and sequenced by Big Dye Terminator v3.1 Cycle Sequencing Kit (Applied Biosystems) using ABI 3100 DNA Sequencer. The nucleotide sequence was compared with those available sequences on the National Center for Biotechnology Information GenBank (NCBI GenBank) Database employing BLASTn through the Basic Local Alignment Search Tool (BLAST). The phylogenetic tree was constructed adopting the Neighbor-Joining tree method using Molecular Evolutionary Genetics Analysis (MEGA-X) and edited by iTOL v5, Interactive Tree Of Life an online tool for the display, annotation, and management of phylogenetic trees.

### Production of bacterial nanocellulose

Seed cultures of both strains were prepared after 2 days of incubation at 30 °C. One milliliter of each was used to inoculate 50 mL HS medium (2% v/v), in 100 mL conical flask, followed by incubation statically for 10 days at 30 °C. The thin film of BNC formed on the surface of each flask was collected, treated with 1 N NaOH, washed with distilled water and dry weight was determined in g/L. The remaining glucose in the media was measured every 2 days over an incubation period of 10 days using ANALYZER A25 Biosystem^30^ to study the correlation between BNC production and glucose consumption.

### Scanning electron microscopy (SEM)

Nanocellulose produced by both strains were examined by scanning electron microscope JEOL JSM-5300 in the Electronic Microscope Unit in Alexandria University.

### X-ray diffraction (XRD)

The XRD diffractogram of samples was obtained on X-ray powder diffraction—XRD-D2 Phaser (Bruker, Germany) in the Central Laboratory, Faculty of Science, Alexandria University using copper X-ray source operating at 30 kV and 10 mA. Scans were collected at 2° per min in the 2θ range of 10°–100°. The crystallite size was estimated using Scherrer’s Eq.^49^:

1 $${\text{CrS }} = K\lambda /\left( {\beta {\cos}\theta } \right)$$

where (k) is the dimensionless Scherrer constant = 0.94, (λ) is the X-ray wavelength = 1.54184 nm, (β) is the peak full width at half maximum in radians, and (θ) is the diffraction angle in radians. The Crystallinity Index CrI (%) was calculated according to the method reported by Zheng et al*.* (2019)^50^ as follows:

2 $${\text{CrI }}\left( \% \right) \, = \, \left( {\left( {{\text{I}}_{{\max}} - {\text{ I}}_{{{\text{am}}}} } \right) \, /{\text{ I}}_{{\max}} } \right) \, \times { 1}00$$

where CrI was calculated from the ratio of the height of the maximum (I_max_) and the height of the minimum (I_am_).

### Thermogravimetric analysis (TGA)

TGA is a thermo-analytical technique used to determine the thermal stability of a material and its fraction of volatile components by monitoring the weight change that occurs when a sample is heated at a constant rate. TGA analysis was performed with a LINSEIS STA PT1000 (Germany) Thermal Analyser in the Central Laboratory, Faculty of Science, Alexandria University. For the thermal decomposition behavior test, cellulose samples were dried at 50 °C for 48 h. Water content was calculated by the following equation^51^:

3 $$\left[ {\left( {W _{t} - W _{0} } \right)/W _{t} } \right] \times 100\%$$

where *W* _0_ and *W* _*t* _represent the weight of dried and wet NCs, respectively.

### Bacterial nanocellulose (BNC) hydrogel preparation

Polyvinyl alcohol (PVA) hydrogel containing BNC was prepared by direct dispersion of the nanofibers in an aqueous PVA solution (20%). The PVA/BNC suspension was subjected to freeze–thaw cycles at − 20 °C. The decrease in the crystallinity of BNC reinforced PVA was compensated by the strong interaction and miscibility between the components. The strength of the gels increased in the order of 1.5 wt% > 0.75 wt% ∼ 3.0 wt% > pure PVA^52^.

### Production of bacterial nanocellulose using industrial and agricultural wastes

**Figure 9:**
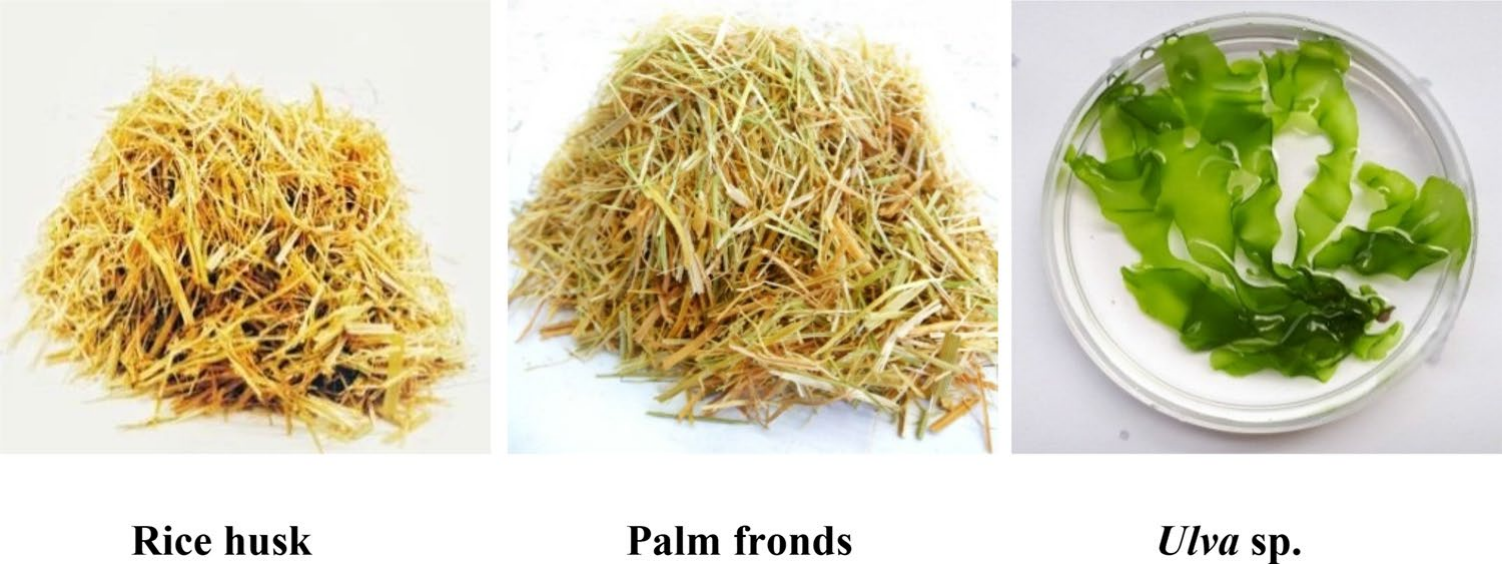
Examples of some examined natural materials.

Waste products such as Palm frond, Sugarcane bagasse, Paper waste, Rice husk, Rice bark, and *Ulva* sp. (Fig. 9) were dried and grounded into powder and subjected to heat treatment followed by acid H_2_SO_4_-heat treatment (see Supplementary Fig. S5) according to Annamalai et al*.*^53^. Molasses was diluted five-fold with distilled water and centrifuged at 6,000 rpm for 20 min to separate solid materials before the acid H_2_SO_4_-heat treatment.

### Statistical analysis

All investigations were performed in three replicates, and the results were statistically analyzed and implemented using XLSTAT Statistical Software, ImageJ 1.48v, and Origin Pro 8.1. The data were analyzed employing ANOVA. The data were carried out based on the values, which were expressed by means ± SE. The significant values were determined at P-value < 0.05, whereas the high significant values were considered at P-value < 0.05, and P-value < 0.001.

## Supplementary information

Supplementary Information.

## Data Availability

The datasets generated and analysed during the current study are available from the corresponding author on reasonable request.
