# Supplementary material for: Novel research on nanocellulose production by a marine Bacillus velezensis strain SMR: a comparative study
Source: Sci Rep. 2020 Aug 26;10:14202. doi: 10.1038/s41598-020-70857-7 (PMC7450066; doi:10.1038/s41598-020-70857-7)
Supplement: Supplementary file 1 — Supplementary Information. [file 41598_2020_70857_MOESM1_ESM.pdf]

Novel research on nanocellulose production by a marine *Bacillus velezensis* strain SMR: a comparative study

Samia S. Abouelkheir[1*], Marwa S. Kamara[2], Salma M. Atia[2], Sara A. Amer[2], Marina I. Youssef[2], Rana S. Abdelkawy[2], Sherine N. Khattab[3], Soraya A. Sabry[4]

[1*]Marine Microbiology Department, Marine Environment Division, National Institute of Oceanography and Fisheries (NIOF), Kayet Bay, El-Anfushy, Alexandria, Egypt. [2] Industrial Microbiology and Applied Chemistry (IMAC) Program, Faculty of Science, Alexandria University, Alexandria, Egypt. [3]Department of Chemistry, Faculty of Science, Alexandria University, Alexandria 21321, Egypt. [4]Botany and Microbiology Department, Faculty of Science, Alexandria University, Alexandria 21321, Egypt.

Corresponding author: Dr. Samia S. Abouelkheir

Researcher of Marine Microbiology,

Marine Environment Division,

National Institute of Oceanography and Fisheries (NIOF), Kayet Bay, El-Anfushy, Alexandria, Egypt

Tel. no.: 02 01122644470

ORCID: https://orcid.org/0000-0001-8559-6787

E. mail address: samiaabouelkhir@yahoo.com, Samiasaad50@gmail.com

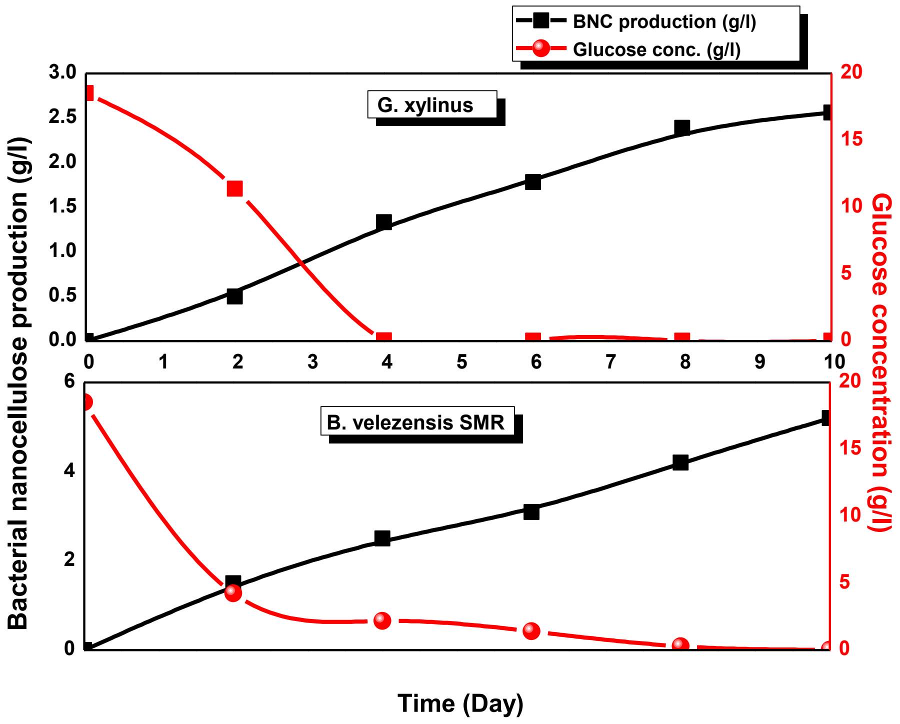

Figure S 1. Graphs showing BNC production and residual glucose concentration by *G. xylinus* and *B. velezensis* SMR.

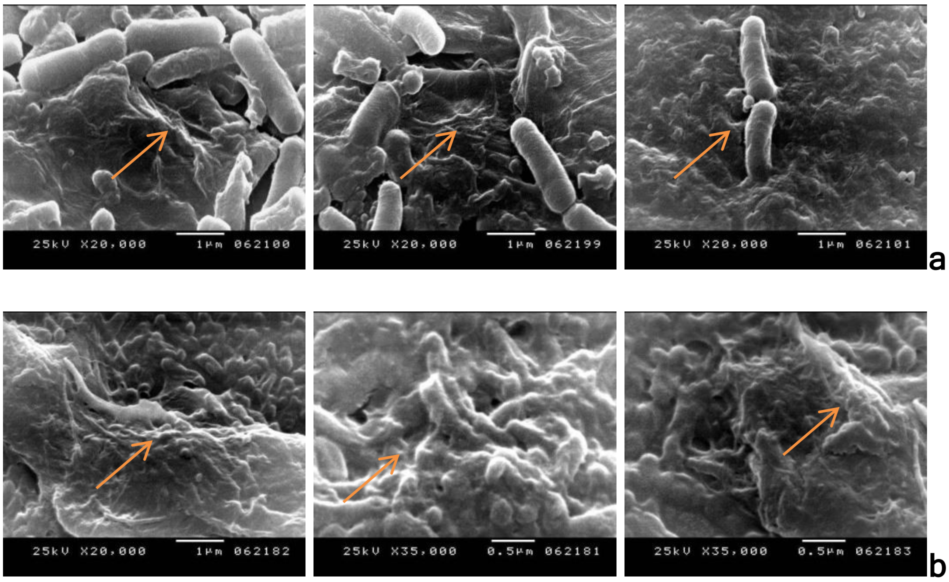

Figure S2. SEM images of BNC fibers obtained after 10 days by *G. xylinus* (a) and *B. velezensis* SMR (b).

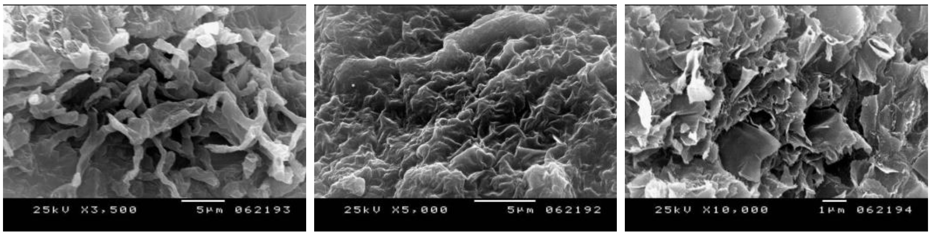

Figure S3. SEM images of bacterial nanocellulose sheet.

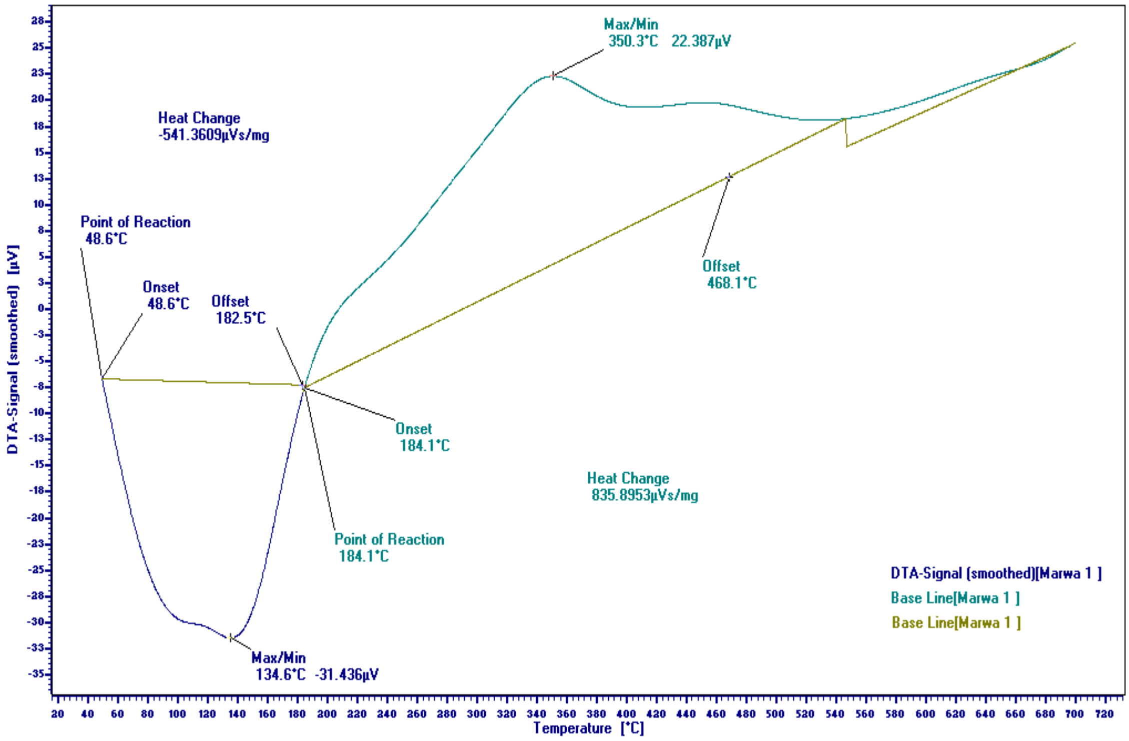
Max/Min
350.3°C 22.387µV
Heat Change
-541.3609µVs/mg
Point of Reaction
48.6°C
Onset
48.6°C
Offset
182.5°C
Offset
468.1°C
Onset
184.1°C
Heat Change
835.8953µVs/mg
Point of Reaction
184.1°C
DTA-Signal (smoothed)[Marwa 1]
Base Line[Marwa 1]
Base Line[Marwa 1]
Max/Min
134.6°C -31.436µV
DTA-Signal (smoothed) [µV]
Temperature [°C]

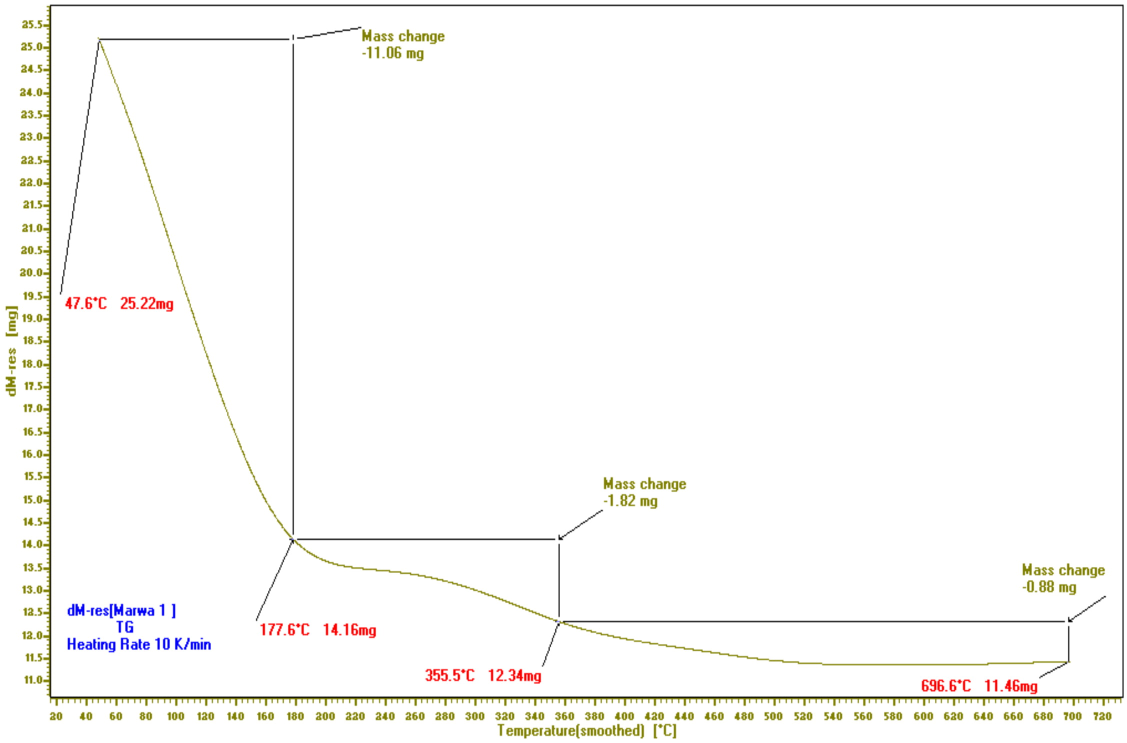
Mass change
-11.06 mg
47.6°C 25.22mg
Mass change
-1.82 mg
Mass change
-0.88 mg
dM-res[Marwa 1]
TG
Heating Rate 10 K/min
177.6°C 14.16mg
355.5°C 12.34mg
696.6°C 11.46mg
dM-res [mg]
Temperature(smoothed) [°C]

a

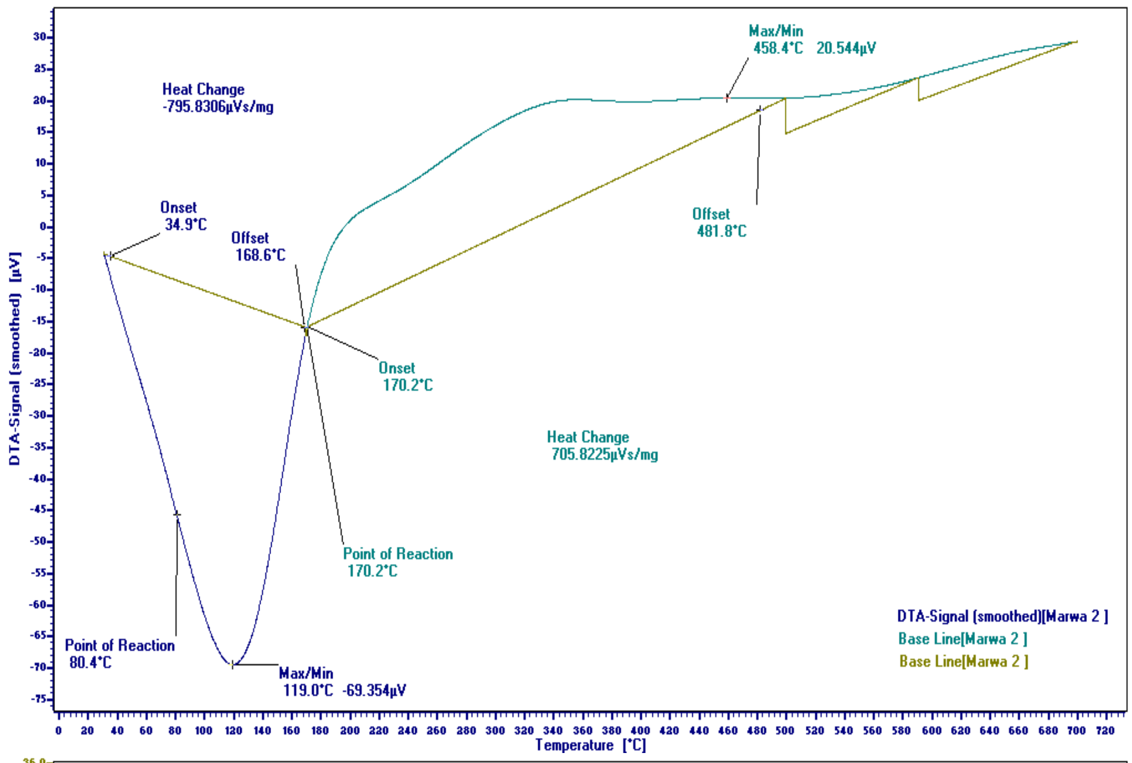
Heat Change
-795.8306µVs/mg
Max/Min
458.4°C 20.544µV
Onset
34.9°C
Offset
168.6°C
Offset
481.8°C
Onset
170.2°C
Heat Change
705.8225µVs/mg
Point of Reaction
170.2°C
Point of Reaction
80.4°C
Max/Min
119.0°C -69.354µV
DTA-Signal (smoothed)[Marwa 2]
Base Line[Marwa 2]
Base Line[Marwa 2]
DTA-Signal (smoothed) [µV]
Temperature [°C]

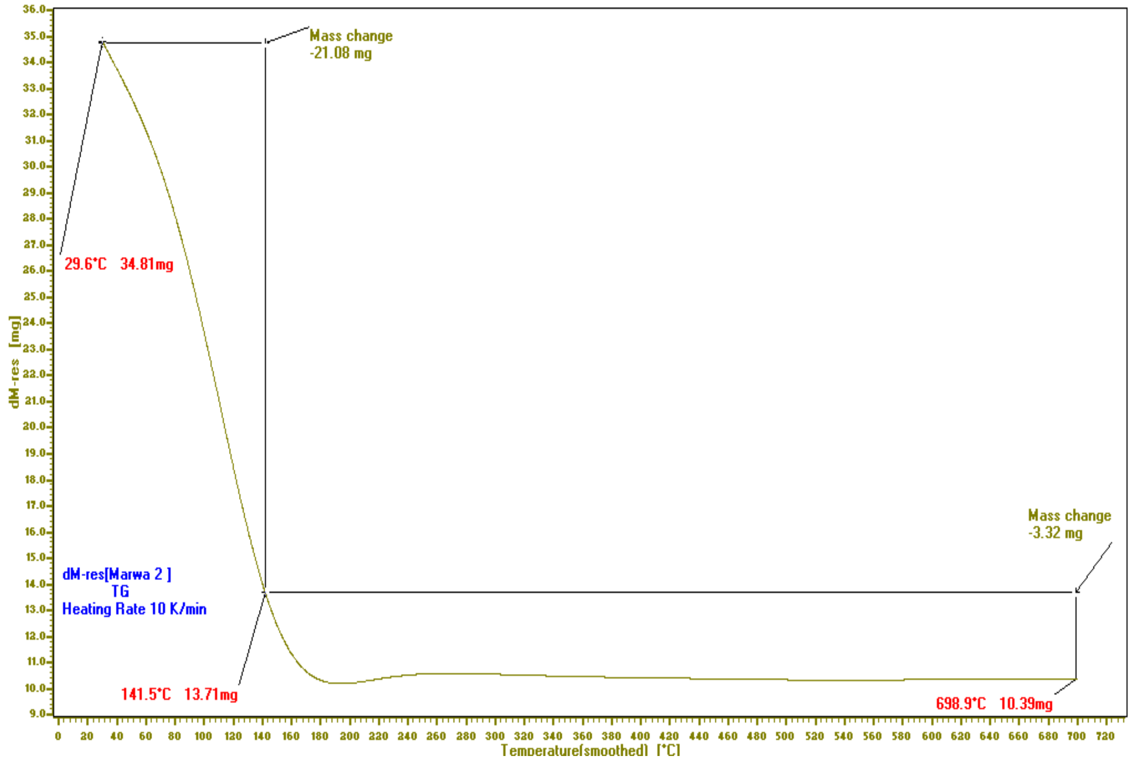
Mass change
-21.08 mg
29.6°C 34.81mg
dM-res[Marwa 2]
TG
Heating Rate 10 K/min
141.5°C 13.71mg
Mass change
-3.32 mg
698.9°C 10.39mg
dM-res [mg]
Temperature(smoothed) [°C]

b

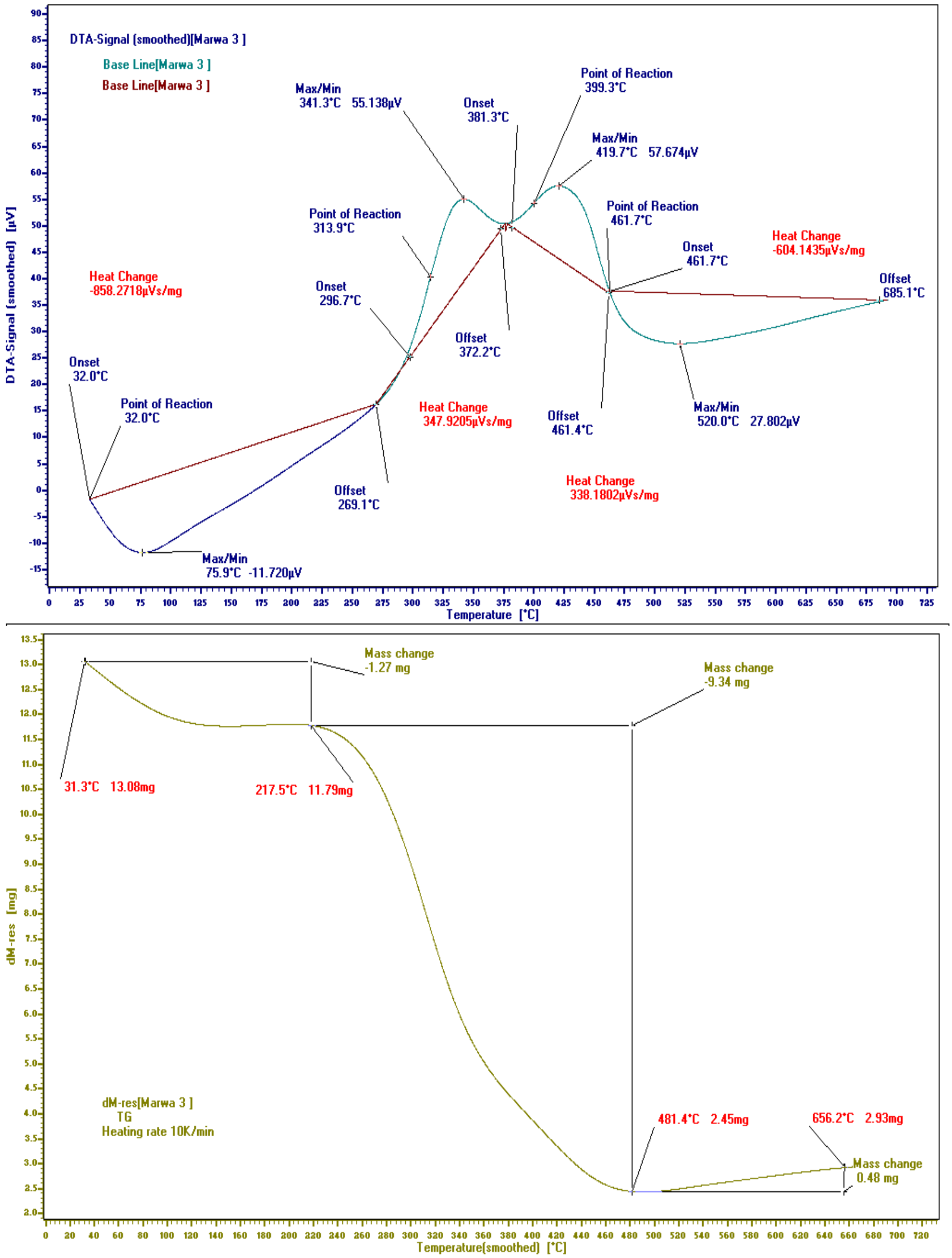

C

Figure S4. DTA and TG curves of NC of *G. xylinus* (a), *B. velezensis* SMR (b), and rice husk (c).

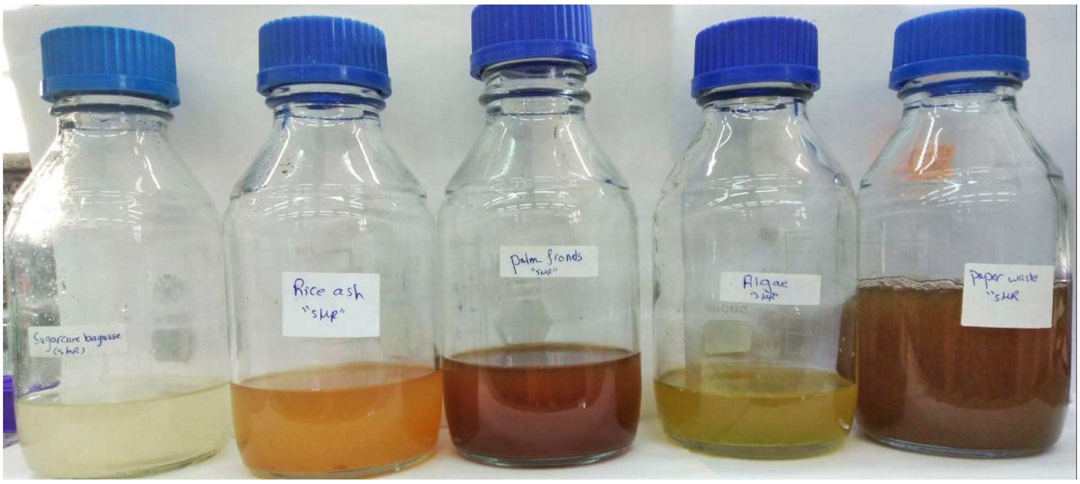

Figure S5. Natural wastes after treatment.
